# High Incidence Is Not High Exposure: What Proportion of Prevention Trial Participants Are Exposed to HIV?

**DOI:** 10.1371/journal.pone.0115528

**Published:** 2015-01-08

**Authors:** Dobromir Dimitrov, Deborah Donnell, Elizabeth R. Brown

**Affiliations:** 1 Vaccine and Infectious Disease and Public Health Sciences Divisions, Fred Hutchinson Cancer Research Center, Seattle, Washington, United States of America; 2 Department of Applied Mathematics, University of Washington, Seattle, Washington, United States of America; 3 Department of Global Health, University of Washington, Seattle, Washington, United States of America; 4 Department of Biostatistics, University of Washington, Seattle, Washington, United States of America; University of Maryland School of Medicine, UNITED STATES

## Abstract

**Objective:**

Randomized clinical trials of HIV prevention in high-risk populations of women often assume that all participants have similar exposure to HIV. However, a substantial fraction of women enrolled in the trial may have no or low exposure to HIV. Our objective was to estimate the proportion of women exposed to HIV throughout a hypothetical high-risk study population.

**Methods:**

A stochastic individual-based model was developed to simulate the sexual behavior and the risk of HIV acquisition for a cohort of sexually active HIV-uninfected women in high HIV prevalence settings. Key behavior and epidemic assumptions in the model were based on published studies on HIV transmission in South Africa. The prevalence of exposure, defined as the proportion of women who have sex with HIV-infected partner, and HIV incidence were evaluated.

**Results:**

Our model projects that in communities with HIV incidence rate of 1 per 100 person years, only 5-6% of women are exposed to HIV annually while in communities with an HIV incidence of 5 per 100 person years 20-25% of women are exposed to HIV. Approximately 70% of the new infections are acquired from partners with asymptomatic HIV.

**Conclusions:**

Mathematical models suggest that a high proportion of women enrolled in HIV prevention trials may be unexposed to HIV even when incidence rates are high. The relationship between HIV exposure and other risk factors should be carefully analyzed when future clinical trials are planned.

## Introduction

The usual statistical design and analysis approaches for clinical trials of HIV prevention in high-risk populations of women assume that all women enrolled in the trial have similar exposure to HIV. Heterogeneity in magnitude and frequency of exposure can lead to biased estimates of effectiveness and reduced power (for a general discussion see [1] and for specific examples in HIV prevention see [2]). This is especially acute when high proportions of participants are unexposed to HIV and therefore not at risk for the study endpoint, HIV infection. When male partners are not monitored in these trials, there are no available measures of participants’ exposure to HIV.

Lingappa et al reported HIV results in participants screened for a study recruiting HIV discordant couples that used recruitment strategies similar to trials that enroll HIV-uninfected women [3]. Across 12 sites in Kenya, Uganda, Tanzania, South Africa, Zambia and Botswana where HIV screening results were available for the male partners, only 14% of the women who would be eligible for a high-risk population prevention trial (HIV seronegative) had partners who were HIV seropositive.

Given the paucity of data about HIV exposure of women enrolled in clinical trials, we designed a modeling study to simulate the sexual behavior and risk of HIV acquisition for a cohort of uninfected women. Our objective was to estimate the proportion of potential clinical trial participants exposed to HIV and the incidence of HIV for given population risk characteristics, such as male HIV prevalence, sexual behavior and concurrency of partnerships.

## Methods

We used stochastic individual-based models to simulate HIV risk for a cohort of 2000 sexually active HIV-uninfected women in a high HIV prevalence setting. The sexual experience of each woman, including partnership formation and dissolution and frequency and type of sex acts, was simulated over a 12-month period in discrete time (units = days). Each day, a woman may acquire new partners, have sex (protected or unprotected) with one or more of her active partners, or terminate an active relationship. The male partners’ characteristics, baseline HIV status and risk of HIV acquisition were simulated according to data-derived parameters. Published research on sexual behavior patterns and published studies on HIV transmission in South Africa [4, 5] informed behavior and epidemic assumptions in the model. The impact of the parameter values on the projected proportion of infected and exposed women was explored in multivariate sensitivity analysis.

### Sexual Behavior

Each woman may be involved in two types of sexual partnerships: i) short-term partnerships with an average duration of 6 months and characterized by higher rates of protected sex; ii) long-term partnerships with an average duration of 10 years and a low rate of protected sex. All new partnerships start as short-term, converting into long-term after 9 months. Following the population structure described by Johnson et al.[4], we divide the women into *low-risk* and *high-risk* groups that define their simulated sexual activity. The *high-risk* women may have up to two concurrent partnerships, one of which may be long-term; while *low-risk* women are serially monogamous. This simplifying assumption allows us to reproduce the partnership distribution representative for South Africa [4, 6] where the majority of women are in stable partnerships while fewer are involved in multiple partnerships with shorter duration.

### Partnership formation and dissolution

Two distinct scenarios of partnership formations were simulated assuming different mixing patterns between risk groups. The main scenario assumed *assortative mixing* with partnerships formed more often between individuals from the same risk groups. In other words, women are more likely to partner with men who have similar risk (high or low). The degree of assortativity (the propensity to choose a partner with similar risk) is representative for the sexual mixing patterns in South Africa [4]. In the second scenario (*proportional mixing*) no preferential pairing of partners from the same risk groups was assumed, i.e., *all* women have equal chance to establish partnerships with high-risk men.

Both scenarios shared sexual behavior assumptions based on data from high HIV prevalence communities [4, 6–8]. New partnerships begin at a fixed rate that is almost halved when women are in active short-term partnerships and reduced 7-fold if in long-term partnerships. The minimal duration of each partnership was fixed at 30 days. The long- (short-) term partnerships dissolve at 5% (80%) average rate annually but the dissolution rate is elevated if another partnership is active at the time. A *low-risk* woman has 2 months on average between the end of one and the beginning of a new relationship compared to 1 month for *high-risk* women. Values of behavioral parameters are specified in Table 1.

**Table 1:** Parameters values used in the main analysis.

**Parameter Description**	**Values and ranges**	**Reference**
**A. Epidemic parameters**		
Female HIV acquisition risk per unprotected vaginal act with short-term partner in asymptomatic HIV stage	0.0065	[4]
Female HIV acquisition risk per unprotected vaginal act with long-term partner in asymptomatic HIV stage	0.0024	[4]
Condom efficacy against HIV per sex act	90%	[15]
Relative risk per sex act with partner in acute HIV compared to asymptomatic HIV stage	9.2	[9]
Relative risk per sex act with partner in late HIV compared to asymptomatic HIV stage	7.3	[9]
Duration of acute HIV stagei	120 days	[16]
Duration of asymptomatic HIV stage	8 years	[16]
Duration of late HIV stage	1 year	[16]
Relative risk per receptive anal compared to vaginal intercourse	10	[10]
HIV-prevalence among male partners	2–18%	Explored
HIV-incidence among male partners	0.2–2%	Explored
**B. Behavioral parameters**		
Average number of sexual acts per year for women in the cohort	68–72	Calculated
Rate of condom use in long-term partnerships	15%	[4]
Rate of condom use in short-term partnerships	40%	[4]
Proportion of partnerships in which anal sex is practiced	20%	[14]
Probability for a sex acts with a partner who practice anal sex to include anal intercourse	40%	[5]
Minimal duration of a partnership	30 days	Assumed
Time to convert from short- to long-term partnership	270 days	Assumed
Proportion of women who are likely to have concurent partnerships (high risk group)	25%	[4]
Proportion of men who are likely to have concurent partnerships (high risk group)	35%	[4]
Degree of assortative mixing between risk groups	0.56	[4]
Daily probability to acquire a partner if not in a partnership for high- (low-) risk women	0.033 (0.017)	[4]
Relative partner acquisition rate for high-risk women who already have a short- (long-) term partner	0.54 (0.17)	[4]
Daily probability to break an active short- (long-) term partnership if not in concurrent partnerships	0.0024 (0.00014)	Assumed
Daily probability to break an active short- (long-) term partnership when in concurrent partnerships	0.0096 (0.00028)	Assumed
Monthly frequency of sex acts in short- (long-) term partnerships	4 (6)	[4]

### Sexual activity within a partnership

The average frequency of sexual acts in a partnership remained constant for the duration of the relationship with coital frequency among married (long-term) couples assumed to be six times per month compared to four times per month for unmarried (short-term) couples. In the 20% of partnerships in which anal intercourse is practiced, an average of 40% of intercourse was assumed to be anal based on data from Kalichman et al. 2009 [5]. The proportion of sex acts protected by condom was assumed to be significantly higher in short-term partnerships (40%) compared to long-term partnerships (15%).

### HIV transmission

All women are initially HIV-negative. The HIV status of their partners is randomly assigned based on assumed HIV prevalence in different risk groups (high and low) of the male population. The HIV acquisition risk per vaginal intercourse was differentiated by the partner’s stage of infection with asymptomatic stage risk fixed at 0.24% (0.65%) for a long-term (short-term) partnership while the multipliers which represent the elevated HIV risk during acute and late stages were taken from published meta-analyses [9]. Anal intercourse was assumed to be 10 times riskier than vaginal intercourse with respect to HIV transmission [10]. The protective efficacy of male condoms against HIV was fixed at 90%. Details on the way HIV risk per sex act is evaluated are included in the Supporting Information (see S1 File).

### Population Characteristics

The number of short- and long-term partnerships for each woman at the start of the simulation is assigned randomly based on demographic data representative of South Africa [4, 6] resulting in 11% of low-risk individuals not currently having a partner and the vast majority of the rest (~60%) having a single long-term partner (see Table A in S1 File). In contrast, high-risk individuals are less likely to have no partners (8%) or have a long-term partner (20%) but more likely to report concurrent partnerships (18%). Behavioral characteristics of the cohort at the end of the simulated period are presented in Table 2.

**Table 2:** Characteristics of the simulated female cohort based on the last month of sexual activity.

Characteristics	Entire cohort	High-risk group	Low-risk group
Average number of sex acts per month	6.1	7.7	5.6
Average number of unprotected sex acts per month	4.9	5.7	4.6
Cohort distribution by number of partners:			
0 partners	1.8%	0.5%	2.2%
1 partner	82.7%	38.3%	97.6%
2+ partners	15.6%	61.2%	0.2%
Women who had anal sex last month	19.7%	25.6%	17.7%

### Population Outcomes of Interest

We focused on two key population outcomes: the prevalence of exposure (PoE) which represents the proportion of women in the cohort who had at least one sexual contact with an infected partner (protected or unprotected) and HIV incidence during the simulated period. Diagram associated with HIV exposure and HIV acquisition is included in the Supporting Information (see S1 File).

## Results

**Figure 1:**
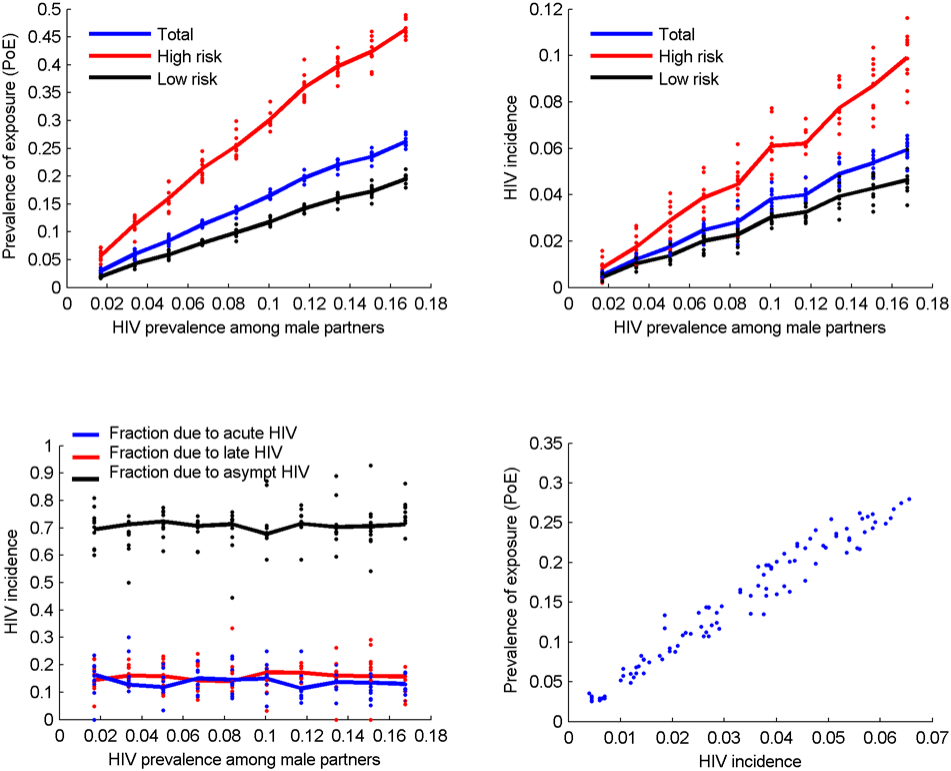
Results from each of the simulations conducted under the assumptions in Table 1 are plotted, illustrating A) prevalence of exposure (PoE) and B) HIV incidence among 2000 originally uninfected women (blue), high-risk group (red) and low-risk group (black) over 1-year period for different values of HIV prevalence among male partners in 10 simulations (dots) per male prevalence level and averaged (thick lines); C) Distribution of female HIV acquisitions over 1-year period by the stage of HIV infection of the transmitting male partner; D) Scatter plot of the infected vs. exposed fractions over 1-year period. All simulations assume assortative mixing between different risk groups when partnerships are formed, i.e. high risk women have greater chance to partner with high risk men and similarly low-risk women partner more often with low-risk men.

We estimate (Fig. 1) the PoE and incidence of HIV infection over a 12 month period given the assumptions in Table 1. Only scenarios with assortative mixing between risk groups are presented in the main text while the proportional mixing scenarios are explored in the Supporting Information. Fig. 1A–1B illustrate the impact of model assumptions on the outcomes of interest. Only 3% of the women are projected to have sexual contact with an HIV-positive partner over 1 year, i.e., PoE = 3%, if the HIV prevalence among male population is 2% (Fig. 1A). PoE increases to 16.5% and 26% when the background prevalence is 10% and 17% respectively. HIV incidence among women varies from 0.5% to 5.9% as HIV prevalence among men varies from 2% to 17% (Fig. 1B). Both PoE and incidence (over 45% and 9%, respectively) are substantially higher among women who are likely to have more than one partner at a time (high-risk group). Fig. 1C shows that approximately 70% of the new infections are acquired from partners with asymptomatic HIV. The remaining 30% are almost equally split between contacts. The contribution of each HIV stage is unaffected by the assumed background HIV prevalence.

Fig. 1D plots PoE against incidence in the first year. Our model projects that 5–6% of the women are exposed to HIV annually in communities with 1% incidence rate compared to 20–25% exposed if the incidence rate is around 5%.

The scenarios with proportional mixing between risk groups project the same overall exposed and infected female fractions but lower PoE (by 3–4%) and incidence (by 1%) among the high-risk group (see Fig B in S1 File).

To examine the robustness of the model to the behavioral and epidemic assumptions, we conducted a sensitivity analysis (detailed in the Supporting Information) using parameter ranges described in Table B in S1 File. Results were largely similar across the range of assumptions, and demonstrated that the PoE could actually be as low as 10% but never higher than 30% when the incidence rate is 5 per 100 person-years. (see Fig. C in S1 File)

## Discussion

HIV prevention effectiveness trials in women are not restricted to participants in serodiscordant partnerships. Instead, women who are sexually active are targeted for recruitment under the assumption that these women will be at risk for HIV. In this modeling study we demonstrated that high incidence rates, consistent with rates in current clinical trials, may be observed even when less that 30% of the women had sex with an HIV-infected man.

### Advantages

The individual-based modeling approach allowed for integrating different patterns of sexual behavior observed in specific population with high HIV prevalence. The incidence rates estimated here are consistent with those observed in recent clinical trials [11–14]. The results of these analyses depend on behavioral and epidemic assumptions employed in the model; our sensitivity analyses demonstrated similar results across the range of parameters, consistently showing a substantial majority of the cohort were not exposed to HIV even in high incidence settings.

### Implications/Recommendations

The implications of enrolling large proportions of HIV-unexposed participants into clinical trials include loss of power and bias when estimating the intervention effect (e.g. hazard ratio of HIV-infection). These bias effects are exacerbated in secondary analyses where HIV exposure may be related to another exposure of interest with unbalanced HIV exposure between the comparison groups. As an example, in a secondary analysis comparing injectable to oral contraception methods, if women who have a known HIV-infected partner are more likely to choose injectable contraception, statistical analyses will indicate an association between injectable contraception use and HIV risk, even when there is no biological connection. Without some reliable measure of known HIV exposure, these analyses become impossible to interpret.

### Conclusion

We have demonstrated that a high proportion of women enrolled in HIV prevention trials may be unexposed to HIV even when incidence rates are high. Results from these trials, especially secondary, must be interpreted with the recognition that not all women are exposed to HIV and that it may be impossible to untangle the relationship between HIV exposure and another risk factor of interest.

## Supporting Information

S1 File Supporting Information. (DOCX)
