# Supplementary material for: High Incidence Is Not High Exposure: What Proportion of Prevention Trial Participants Are Exposed to HIV?
Source: PLoS One. 2015 Jan 8;10(1):e0115528. doi: 10.1371/journal.pone.0115528 (PMC4287619; doi:10.1371/journal.pone.0115528)
Supplement: S1 File — (DOCX) [file pone.0115528.s001.docx]

**Supporting Information**

**High incidence is not high exposure: What proportion of prevention trial participants are exposed to HIV?**

**1. HIV risk per sex act**

The probability of HIV acquisition per act with infected partner is determined based on the type of the act (vaginal vs. anal), if the act is protected by condom as follows:

$$P= (1-c\alpha_{c})r^{A}R\beta$$

where:

$c$ - condom use variable (c=0 for unprotected, c=1 for protected act)

$\alpha_{c}$ - condom efficacy per act

A – variable representing the type of act (A=0 for vaginal, A=1 for anal act)

*r* - relative HIV acquisition risk per anal act compared to vaginal act

*R* - relative HIV acquisition risk by the stage of HIV infection of the infected partner (R=9.2 for acute, R=1 for asymptomatic, R=7.3 for late HIV stage)

*β*- HIV acquisition risk per unprotected vaginal act with infected partner in asymptomatic HIV stage

**2. Decision-tree diagram for HIV exposure and HIV risk**


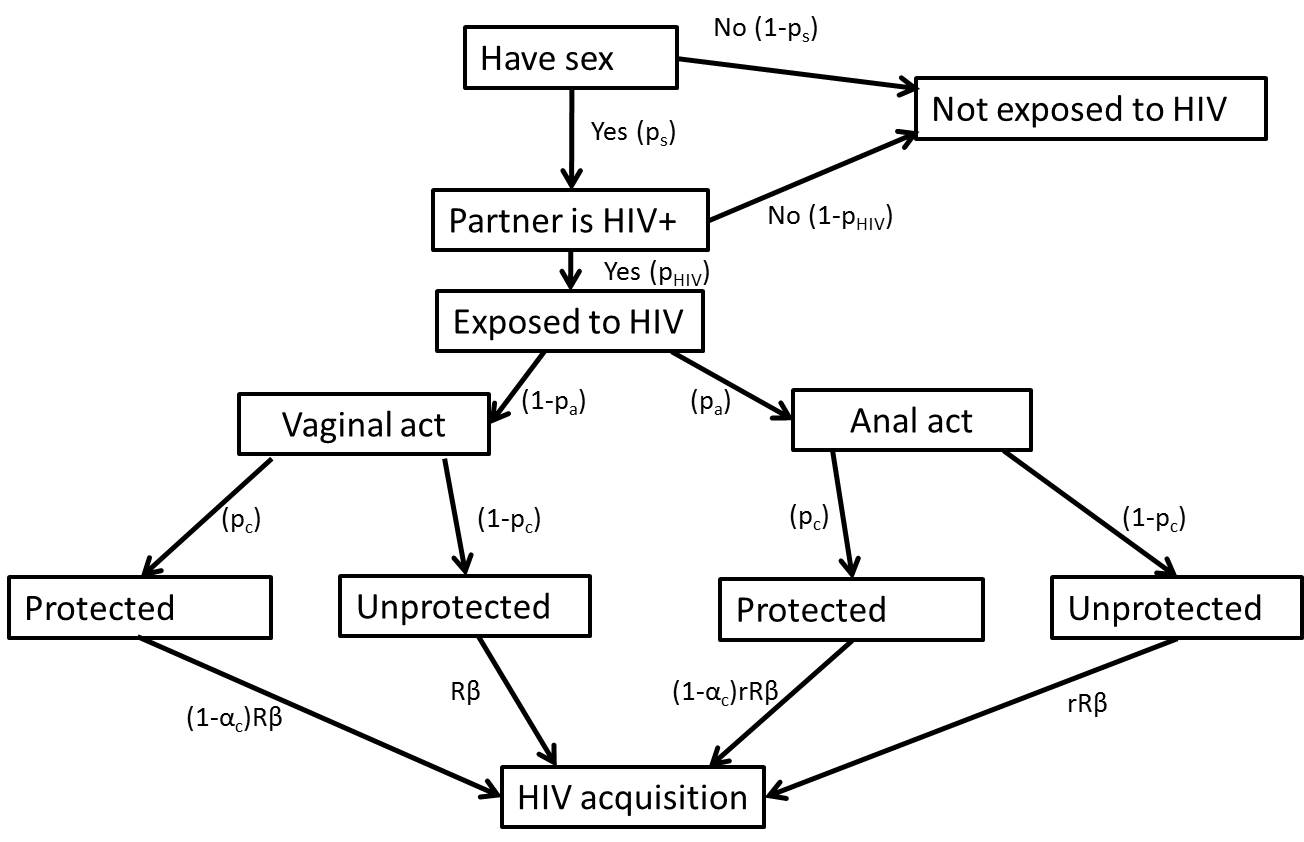


The probability of each transition is given next to the arrow and depends on the probabilities to have sex (p_s_), the sexual act to be with infected partner (p_hiv_), to have anal sex (p_a_) and use condom (p_c_). Those probabilities are determined from the parameters in Table 1 as follows:

- probability to have sex (p_s_) depends on the frequency of sex acts with the active partners
- probability the act to be with infected partner (p_hiv_) depends on the HIV prevalence among male partners which influences the initial distribution of the partners and the HIV status of newly acquired partners. (p_hiv_) also depends on the HIV incidence among male partners which influences the risk that an uninfected partner may acquire HIV outside the partnership.
- probability to have anal sex act (p_a_) depends on the proportion of the male partners who practice anal sex and on the proportion of anal sex acts in such partnerships
- probability of condom use (p_c_) depend on the type of partnership (long- vs. short-term)

The probability of HIV acquisition is governed by the formula of the HIV risk per act described above.

**3. Simulation procedure and bookkeeping**

1. 2000 women are created and assigned with risk group, number and type of current partnerships.
2. Existing partnerships are initialized with the following attributes:
   - starting day of the partnership with respect to the start of the simulation. All long-term partnerships are assumed an year old (starting day = -365 ) while short-term partnerships start between 90 and 210 days prior the simulation
   - partner’s risk level (high or low). 35% of male population is assumed high-risk.
   - Frequency of sexual activity
   - Daily probability to break up
   - Current HIV status of the partner
   - Practicing anal sex (yes, no). 20% of male partners are assumed to practice anal sex
3. Sexual activity is simulated for a year and all sexual acts are recorded. Daily each participant may :
   - Initiate a new partnership
   - Have sex with some of her current partners based on the frequency of acts for each partnership. Probability of condom use and HIV acquisition risk per act depend on the type of partnership. HIV transmission may occur if the woman is HIV- and her partner is HIV+. The probability of HIV acquisition depends on the type of the act (vaginal vs. anal) and if the act is protected by condom (see the risk formula and diagram above).
   - Active partner(s) may acquire HIV outside the relationship depending on his risk level
   - Short-term partnership convert into long-term when 9 months limit is reached
   - Break up a partnership. That depends on the type of the partnership and the current concurrency status. May be forced if short-term partnership reaches 9 months but another long-term partnership is in place.

All sexual acts are recorded. Each record consists of:

- Participant identifier
- Day of the act
- Partner identifier
- Current partnership type (long- vs. short-term)
- Risk level of the partner
- HIV status of the partner. If infected the day of HIV acquisition is recorded.
- Type of the sex act (vaginal vs. anal)
- Use of protection
- If the sex act results in HIV transmission

**4. Mixing patterns**

The probability for a high-risk woman to acquire a partner from the high-risk group is:

(1-ε)+ ε * (proportion of high-risk men)

The probability for a low-risk woman to acquire a partner from the high-risk group is:

ε * (proportion of high-risk men)

The degree of assortative mixing (ε) takes values between 0 and 1 and control the level of preferential pairing between partners from the same risk groups.

We explore two mixing scenarios:

- Proportional mixing (ε=1) in which each woman has the same probability to initiate partnership with man from the high risk group regardless of her risk status. The probability for a new partnership to be with a high-risk man is equal to the male HIV prevalence.

Assortative mixing (ε=0.56) in which women from high-risk group have greater chance to pair with men from high risk group compared to women from the low-risk group.

**Table A.** Targeted distribution per risk group with respect to existing partnerships by type (short- and long-term)

| Risk group | No partners | 1 short-term | 2 short-term | 1 long-term | 1 long-term  1 short-term |
| --- | --- | --- | --- | --- | --- |
| High-risk | 8% | 60% | 12% | 14% | 6% |
| Low-risk | 11% | 29% | 0% | 60% | 0% |

**5. Additional results**


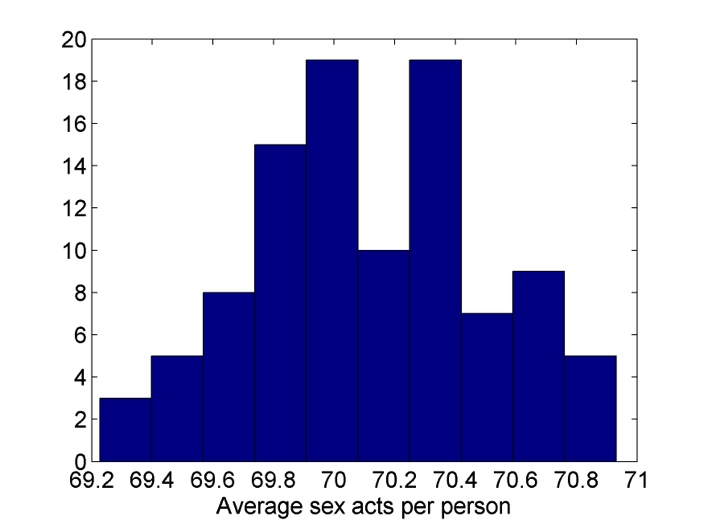

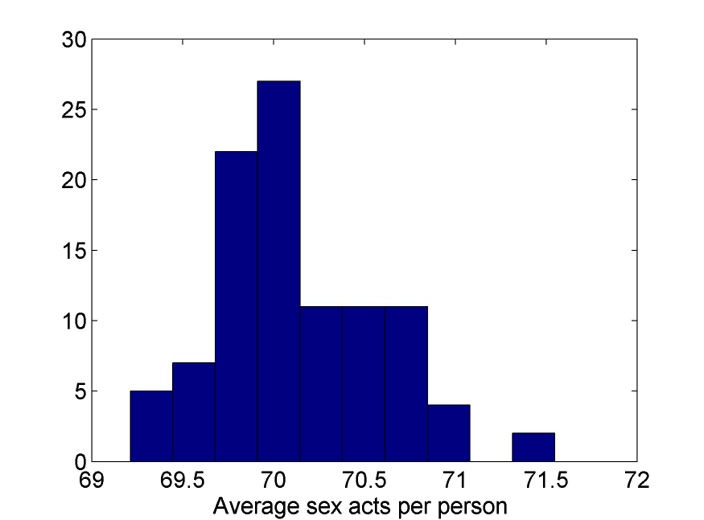


A)

B)

**Figure A. Histograms of the average number of sex acts per year that women have in 100 simulations assuming A) assortative mixing between different risk groups when partnerships are formed, i.e. high risk women have the greater chance to partner with high risk men and similarly low-risk women partner more often with low-risk men and B) proportional mixing between different risk groups when partnerships are formed, i.e. high and low risk women have the same chance to partner with high risk men.**


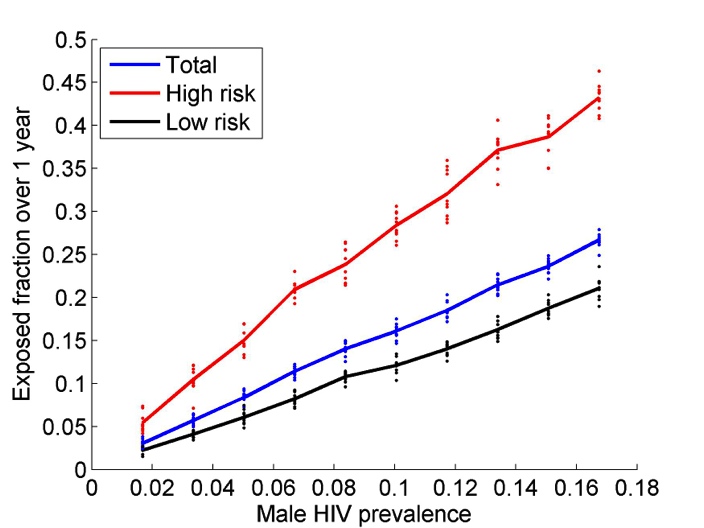

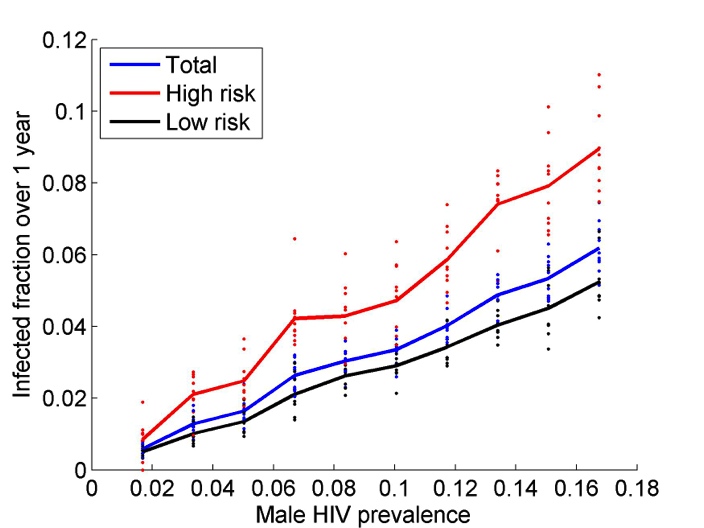

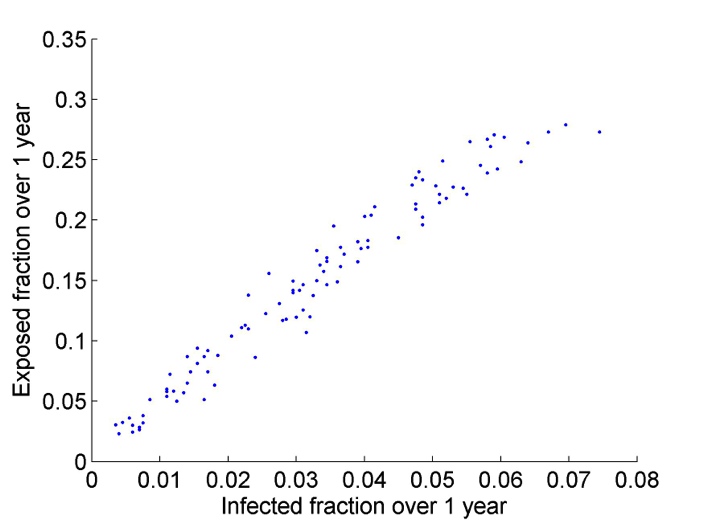

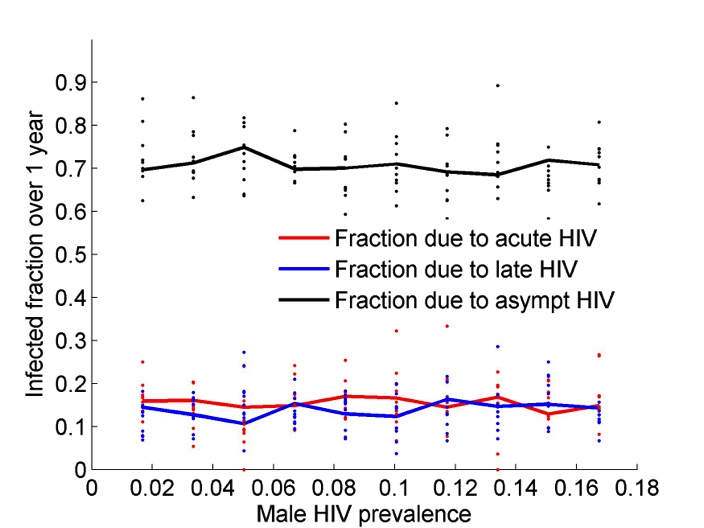


A)

B)

C)

D)

**Figure B. A) Exposed and B) infected fractions of 2000 originally uninfected women (blue), high-risk group (red) and low-risk group (black) over 1-year period for different values of HIV prevalence among male partners in 10 simulations (dots) per male prevalence level and averaged (thick lines); C) Scatter plot of the infected vs. exposed fractions over 1-year period; D) Distribution of female HIV acquisitions over 1-year period by the stage of HIV infection of the transmitting male partner. All simulations assume proportional mixing between different risk groups when partnerships are formed, i.e. high and low risk women have the same chance to partner with high risk men.**

**5. Sensitivity analysis**

We have simulated the sexual behavior and the risk of HIV acquisition for a cohort of 2000 sexually active HIV-uninfected women over 1 year using 1000 randomly sampled sets of 25 model parameters to study the sensitivity of our results to specific epidemic and behavioral assumptions. List of parameter ranges and results are presented below.

**Table B.** Parameters ranges used in the sensitivity analysis

| **Parameter Description** | **Ranges** |
| --- | --- |
| 1. **Epidemic parameters** |  |
| Female HIV acquisition risk per unprotected vaginal act with long-term partner in asymptomatic HIV stage | 0.002-0.003 |
| Relative female HIV acquisition risk per unprotected vaginal act with short-term partner in asymptomatic HIV stage | 1-3 |
| Condom efficacy against HIV per sex act | 80%-95% |
| Relative risk per sex act with partner in acute HIV compared to asymptomatic HIV stage | 4.5-20 |
| Relative risk per sex act with partner in late HIV compared to asymptomatic HIV stage | 4.5-12 |
| Duration of acute HIV stage | 120 days |
| Duration of asymptomatic HIV stage | 8 years |
| Duration of late HIV stage | 1 year |
| Relative risk per receptive anal compared to vaginal intercourse | 5-20 |
| HIV prevalence, high-risk men | 1%-20% |
| Relative HIV prevalence, low-risk men | 0.5-1 |
| HIV incidence to prevalence ratio | 0.08-0.12 |
| 1. **Behavioral parameters** |  |
| Monthly frequiency of sex acts in long-term partnerships | 4-8 |
| Relative frequiency of sex acts in short-term partnerships | 0.5-1.5 |
| Rate of condom use in long-term partnerships | 10%-20% |
| Rate of condom use in short-term partnerships | 20%-60% |
| Proportion of partnerships in which anal sex is practiced | 0-40% |
| Probability for a sex acts with a partner who practice anal sex to include anal intercourse | 20-60% |
| Minimal duration of a partnership | 30 days |
| Time to convert from short- to long-term partnership | 270 days |
| Proportion of women who are likely to have concurent partnerships (high-risk group) | 10-40% |
| Proportion of men who are likely to have concurent partnerships (high-risk group) | 20-60% |
| Degree of assortative mixing between risk groups | 0-1 |
| Daily probability to acquire a partner if not in a partnership for low-risk women | 0.01-0.03 |
| Relative probability to acquire a partner if not in a partnership for high-risk women | 1-3 |
| Relative partner acquisition rate for high-risk women who already have a short- term partner | 0.3-0.7 |
| Relative partner acquisition rate for high-risk women who already have a long- term partner | 0.1-0.3 |
| Daily probability to break an active long-term partnership if not in concurrent partnerships | 0.00009-0.00027 |
| Daily probability to break an active short-term partnership if not in concurrent partnerships | 0.0012 |
| Relative break up rate for long-term partnerships when in concurrent partnerships | 1-10 |
| Relative break up rate for short-term partnerships when in concurrent partnerships | 1-4 |


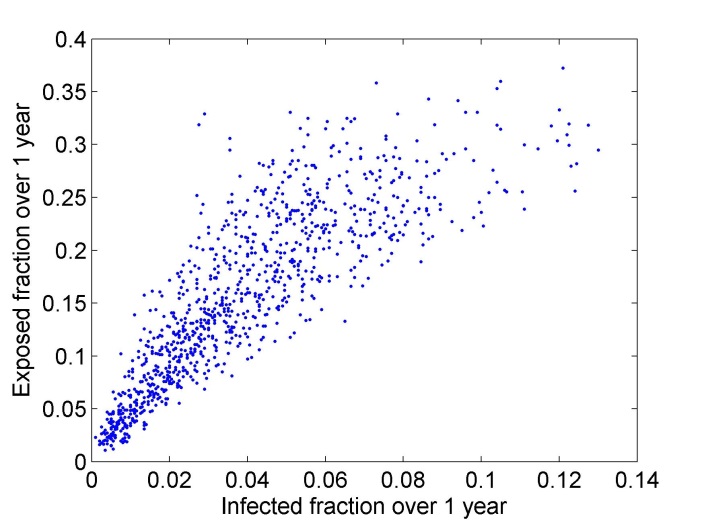

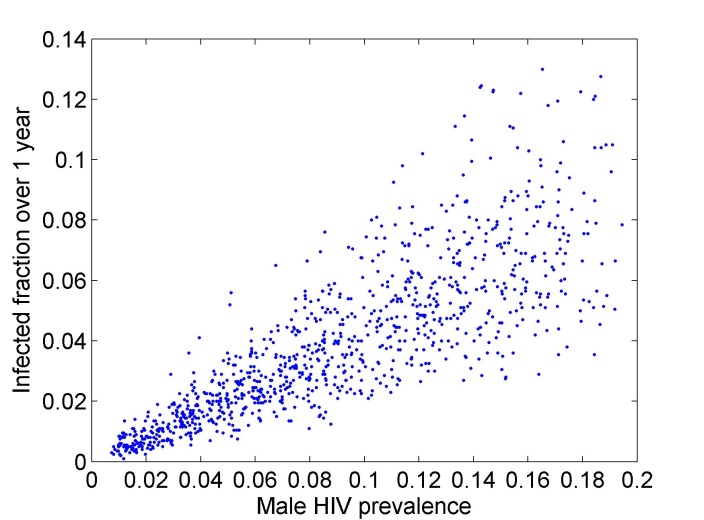

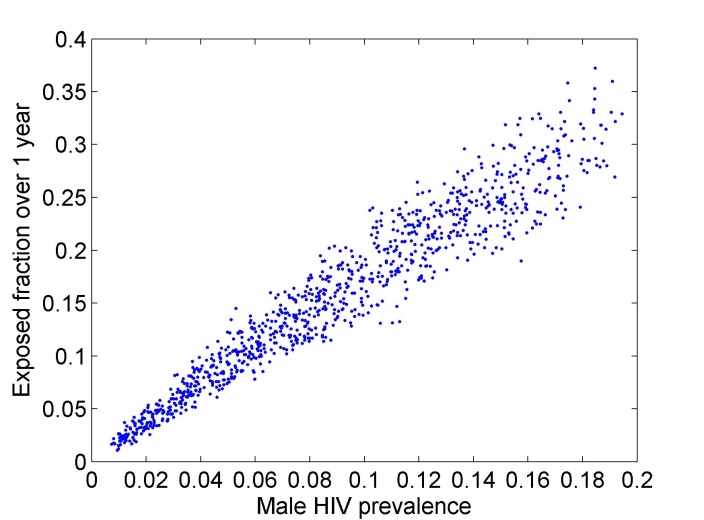


A)

B)

C)

**Figure C. Scatter plot of A) the exposed and B) the infected fractions and C) the infected vs. exposed fractions of 2000 originally uninfected women over 1-year period based on 1000 sets of randomly selected parameters. All simulations assume assortative mixing between different risk groups when partnerships are formed, i.e. high risk women have the greater chance to partner with high risk men and similarly low-risk women partner more often with low-risk men.**
